# A device for production of PAGAT dosimetry gel

**DOI:** 10.1002/mp.70079

**Published:** 2025-11-08

**Authors:** Christina Stengl, Maike Kleiss, Ronald E. Teto, Raquel F. Augusto, Christian P. Karger, Armin Runz

**Affiliations:** ^1^ Department of Radiation Oncology Heidelberg University Hospital (UKHD) Heidelberg Germany; ^2^ Division of Medical Physics in Radiation Oncology German Cancer Research Center (DKFZ) Heidelberg Germany; ^3^ Heidelberg Institute for Radiation Oncology (HIRO) National Center for Radiation Research in Oncology (NCRO) Heidelberg Germany

**Keywords:** gel dosimetry, gel preparation device, PAGAT gel

## Abstract

**Background:**

PAGAT (PolyAcrylamide Gelatine gel fabricated at ATmospheric conditions) gel dosimetry provides a valuable three‐dimensional (3D) measurement tool for photon therapy, particularly in the context of advanced radiotherapy techniques such as volumetric modulated arc therapy or intensity‐modulated radiation therapy. However, the production of PAGAT gel is highly sensitive to variations in the preparation process, particularly in open environments.

**Methods:**

To ensure reliable production of PAGAT gel and a simplified workflow for inexperienced users, a gel preparation device was designed. This device enables (i) single‐component preparation, (ii) transfer of gel components, and (iii) filling of gel in 3D‐printed vials. The gel batches prepared with this device were analyzed for stability and reproducibility. Furthermore, sensitivity to oxygen exposure was tested with and without Tetrakis(hydroxymethyl)phosphonium chloride (THPC). Additionally, vials used for gel storage and irradiation were fabricated using two different PolyJet 3D printers, with either internal support material (opaque finish) or no internal support material (glossy finish). Post‐processing of the printed vials was carried out using either sodium hydroxide (NaOH) cleaning or water rinsing.

**Results:**

The PAGAT gel produced with this device demonstrated high reproducibility, with a standard error of the mean below 1.8%. The gel remained stable for up to 14 days post‐preparation. However, THPC was necessary to remove the residual oxygen from the solution. While the choice of 3D printer did not influence gel performance, the cleaning method played a critical role, with NaOH‐cleaned opaque prints showing reduced gel response.

**Conclusions:**

The gel preparation device enabled low‐cost PAGAT gel production. The system provided a stable and closed environment to ensure consistent gel properties, reducing variability in dosimetry applications. Its design also offers potential for use with other polymer gel formulations requiring controlled conditions.

## 1 INTRODUCTION

Accurately quantifying the absorbed dose is a fundamental requirement in radiotherapy. Conventional dosimetry tools, such as ionization chambers, diodes, thermoluminescence detectors (1D), radiochromic films or detector arrays (2D), provide valuable information but are inherently limited to point‐based or planar measurements. These approaches are often insufficient for verifying the complex, three‐dimensional (3D) dose distributions generated by advanced treatment modalities such as Intensity‐Modulated Radiation Therapy and Volumetric Modulated Arc Therapy. These methods involve complex‐shaped 3D dose distributions, and any deviation can result in either insufficient dosing of the tumor or excessive exposure to surrounding healthy tissue.^1^ To minimize related risks, it is essential to determine the 3D dose distribution in a tissue‐equivalent material prior to treatment.^2^ Dosimetric gels offer a possible solution for this purpose, allowing comprehensive 3D dose measurements with tissue‐equivalent material. One of the most used dosimetry gels is polymer gels, which mainly contain water, gelatin, and different monomers that polymerize upon irradiation.^3^ The fundamental mechanism of these gels is based on radiation‐induced polymerization of monomers. This polymerization process is initiated by free radicals produced through the radiolysis of water, leading to the formation of a gel that can effectively measure radiation dose distributions. The resulting polymers are sufficiently large to prevent diffusion, thereby preserving the spatial distribution of dose information within the gel.^4^ While many different polymer gels have been proposed, PAGAT dosimetry gel is often used.^5^

Despite advancements in research over recent decades, dosimetric gels have not yet gained widespread clinical implementation. Commercially available gels offer convenience but are expensive for routine use. In contrast, in‐house gel preparation is more cost‐effective but involves a complex and sensitive process. The dose response is highly susceptible to variations in oxygen concentration, and precise control over parameters like temperature and mixing is required. These factors introduce uncertainties and make consistent gel production challenging, particularly in facilities without prior experience. Although it is possible to prepare polymer gel in an atmospheric environment,^6^ the reproducibility of the gel response is not always warranted. Careful process optimization and quality assurance measures are needed to ensure reproducible results. For both commercial and in‐house produced dosimetry gels, labor‐intensive MRI evaluation is necessary, which may be done by the gel‐supplying company or in‐house by the medical physicist if access to MRI is possible.

In this study, we present a novel, low‐cost, and user‐friendly device designed to support the preparation of dosimetric gels. The system offers a closed and controlled environment that enables continuous temperature monitoring, simplifies component mixing, and facilitates consistent filling of the final gel into containers.

## 2 METHODS

The gel production procedure is based on the approach described by Elter et al.,^6^ but has been significantly advanced through the development of a novel, custom‐built gel production device. This system enables monitoring of critical parameters such as temperature and allows for reproducible in‐house synthesis of PAGAT gel under standardized conditions.

### 2.1 General gel cooking procedure

**FIGURE 1:**
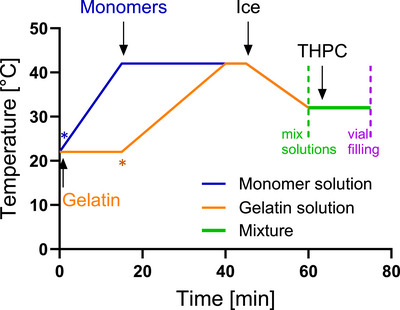
Temperature profiles during the gel cooking process. Gelatin is allowed to swell in water for 15 min at room temperature. The temperature is then raised to 42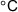 for liquefaction (orange). Water for monomers is heated to 42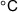, followed by the addition of monomers, which are left to dissolve (blue). After 45 min, ice is added to the device to cool both reactors, and the transfer of the monomers to the gelatin is introduced at 60 min (green). Finally, THPC is added. After further mixing, the prepared vials are filled with the gel (purple) and stored at 4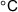 upon irradiation. The blue and the orange stars indicate the time at which the solutions were placed in the gel preparation device.

The gel production process consisted of three steps: (i) monomer and gelatin preparation; (ii) mixing of these two components; (iii) filling into desired vials. The exact amounts of the chemicals is given in Table 1, and the procedure, including temperature control, is visualized in Figure 1.

**TABLE 1:** Components to produce an amount of 1000 g PAGAT gel.

Components	Amount [g]
Gelatin	60
Water _Gelatin Solution_	356
Acrylamide	25
Bis‐Acrylamide	25
Water _Monomer Solution_	534
THPC [μ l]	710
Total Weight	1000

**Step (i)**: Gelatin (from porcine skin, Type A, Sigma‐Aldrich, St. Louis, MO, USA, CAS: 9000‐70‐8) was added to HPLC‐grade water (Hipersolv Chromanorm, Avantor, Radnor, PA, USA; CAS: 7732‐18‐5) and allowed to swell for 15 min. In the meantime, the monomers include Acrylamide (99 %, Sigma‐Aldrich, St. Louis, MO, USA; CAS: 79‐06‐1) and N,N'‐Methylenebisacrylamide (99.5 %, Sigma‐Aldrich, St. Louis, MO, USA, CAS: 110‐26‐9) were mixed and heated up to 42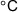 for 30 min to dissolve. Next, the gelatin solution was heated up to 42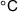. Afterward, the solutions were cooled down to 32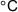.

**Step (ii)**: The monomer solution was transferred to the gelatin solution. Subsequently, Tetrakis(hydroxymethyl)phosphonium chloride (THPC), (80 % solution in water, Sigma‐Aldrich, St. Louis, MO, USA) was added to bind any residual oxygen molecules, and the gel was mixed for 10 min.

**Step (iii)**: The gel was then filled into a container, shielded from light, and stored at 4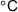 until irradiation.

### 2.2 Gel preparation device

**FIGURE 2:**
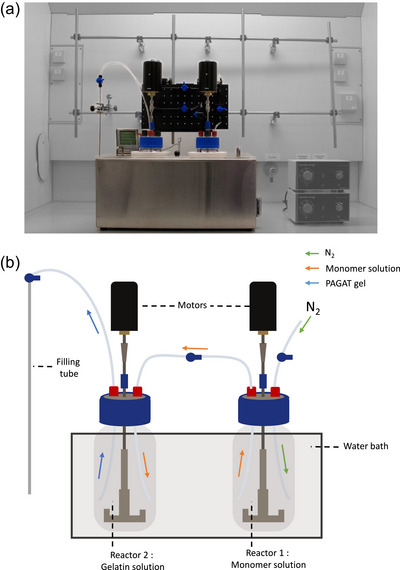
Picture (a) and schematic drawing (b) of the dosimetry gel production device and its components.

The novel gel preparation device streamlines the production and handling of PAGAT gel, enabling a user‐friendly and cost‐efficient manufacturing process. According to the desired batch size, 500 to 2000 mL beakers (DURAN, DWK Life Sciences, Wertheim, Germany), called reactors, can be used to separately prepare the two solutions, the gelatin mixture and monomer mixture. In this study, a 1000 mL reactor was used for the monomer mixture, and a 2000 mL reactor was used for the gelatin mixture. These sizes were chosen to ensure sufficient space in reactor 2 when both solutions are combined. To heat the solutions, a water bath (GGM Gastro International GmbH, Ochtrup, Germany) was filled with 10 liters of water. As shown in the temperature profiles, the reactor of monomers was added to the water bath at timepoint 0 min, while the reactor with gelatin was added at 15 min. A custom‐made lid with openings to accommodate the reactors was designed to retain heat. To measure the temperature, a multi‐sensor temperature gauge (TP17H, ThermoPro, Toronto, Canada) was installed, and a sensor was introduced in each of the reactors and the water bath. To stir the solutions, a laboratory stirrer (SBS‐ER‐3000, Steinberg Systems, expondo GmbH, Berlin, Germany) and motor with a power output of 100 W and a maximum rotation speed of 3400 rpm was selected. To facilitate the transfer process of monomer solution into the gelatin solution, the two reactors were connected via chemically resistant polyurethane tubes (Festo Vertrieb GmbH & Co.KG, Esslingen, Germany) with an inner diameter of 4 mm and an outer diameter of 6 mm. Safety valves with a maximum operating pressure of 10 bar and an inner diameter of 6 mm were installed at every tubing branch to enable opening and closing of the system (DM‐Fit AHUC0606M Standard Ball Valve, 10 bar, Conrad Electronic SE, Mannheim, Germany). To reduce the temperature of the two solutions, a total of 1500 g of ice was added to the water bath. Transfer of the monomer solution into the gelatin solution was achieved within approximately 1 min by applying $N_{2}$ pressure to the reactor holding the monomer solution. After stirring the mixed solution for 10 min, it was filled into the desired container via the same chemically resistant tubing using nitrogen pressure (Figure 2). The total cost of the gel preparation device was $1500 at the time of construction.

### 2.3 Gel irradiation

For photon irradiation, the gel was brought to room temperature 4 h before the measurement. Gel batches were irradiated at a 6 MV linear accelerator (ETHOS, Varian, Palo Alto, USA) of the German Cancer Research Center. The 3D‐printed containers were positioned at the isocenter within 30 × 30 ${\mathrm{cm}}^{2}$ RW3 plates, specifically designed to hold them securely. 10 cm of RW3 was added above the gel vial to simulate a tissue‐equivalent absorber. Reproducibility was assessed by uniformly irradiating vials of three independent gel batches with doses of 0, 1, 3, 5, and 7 Gy. Additionally, stability was evaluated by irradiating vials at 1, 2, 7, and 14 days after preparation with the same doses. To investigate the influence of residual oxygen on the gel response, vials were prepared both with and without THPC, and irradiated with 0, 1, 3, 5, and 7 Gy. After the irradiation, samples were cooled to 4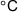 until MRI measurements.

MRI was performed with the Siemens Magnetom Sola 1.5T XQ Numaris/X VA51A‐02ZK (Siemens AG, Munich, Germany) 24 h post‐irradiation using a T2 multi‐echo spin‐echo sequence with a resolution of (1.0 × 1.0 × 0.7) ${\mathrm{mm}}^{3}$, using 32 echoes with an initial echo time of 22.5 ms and an echo spacing of 22.5 ms at a sample temperature of 22 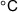. To determine the spin–spin relaxation rate (R2 value) value the reciprocal of the fitted T2 time was analyzed using the Medical Imaging Interaction Toolkit (MITK).^7^

The gel containers were custom‐designed and fabricated using PolyJet 3D printing to achieve a precise, tailored shape. Each container had a cylindrical design with an inner diameter of 25 mm, a height of 51 mm, and a wall thickness of 1 mm. A conical opening with an M5 screw thread and sealing was integrated to facilitate bubble‐free filling. Two 3D printers, the J55 (Stratasys, Israel) and the Objet500 (Stratasys, Israel), were tested using VeroClear (Stratasys, Israel) as printing material. Printing was performed using two finishes: glossy, where no support material was used inside the container, and opaque, where support material was printed within the container. After printing was completed, the support material was removed using either NAOH or pure water. The impact of these two cleaning methods on the gel response was evaluated. For this, the different container sets were filled with the gel and irradiated with 5 Gy, and the influence of the printer, the support structure inside the vial, and the cleaning method was compared.

## 3 RESULTS

### 3.1 Reproducibility and stability of gel preparation

**FIGURE 3:**
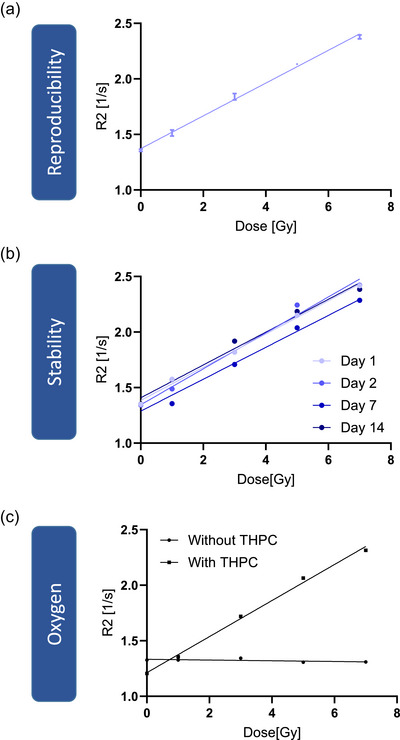
Reproducibility of PAGAT gel for three independent measurements. Data show mean and the standard deviation (a). Stability of PAGAT gel over time (b). Influence of oxygen on the dose response of the gel (c).

Irradiating the gel with 0, 1, 3, 5, and 7 Gy revealed a standard deviation of the mean R2 value over the three batches from 0.3 to 1.8 (mean 1.1 %), indicating a highly reproducible PAGAT gel production with the developed device. A linear fit resulted in a slope of (0.148 ±0.003) (Gys)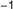 and an *R* 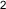 of 0.995 (Figure 3a). Testing the stability of the gel over 14 days after preparation revealed no systematic signal loss. The mean standard deviation of the dose value was 3.7 % (Figure 3b). Furthermore, the impact of residual oxygen on the dose‐response of the gel was tested by omitting the oxygen scavenger THPC. Without THPC the gel did not respond to increasing dose, revealing a reduction of R2 by about 43 % at 7 Gy (Figure 3c).

### 3.2 Impact of customized vial processing with 3D printing

**FIGURE 4:**
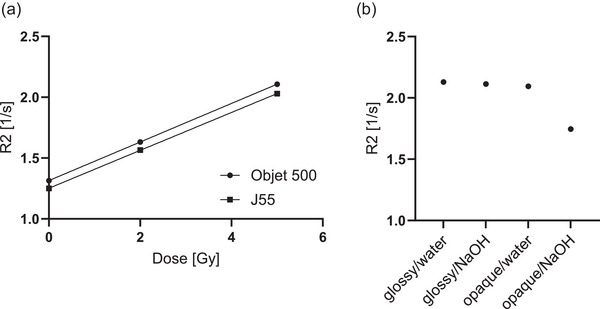
Influence of 3D printer using VeroClear as printing material (a). Impact of the 3D printer and cleaning process when printing the gel vial glossy or opaque and cleaning it with NaOH or with water only (b).

The choice of 3D printer did not affect the gel response. The linear fits resulted in slopes of 0.159 and 0.156 (Gys)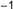 for the Objet500 and the J55, respectively (Figure 4a). However, for vials printed with internal support material (opaque), the cleaning method significantly affected the outcome. Cleaning opaque vials with NaOH reduced the R2 value by approximately 18.5 % compared to water cleaning or using glossy‐printed vials cleaned with NaOH (Figure 4b).

### 3.3 Comparison with manual gel preparation

The improvements and advantages introduced by the device are described in Table 2, which provides a comparison between the manual preparation method, as previously described in Elter et al.,^6^ and our device‐assisted method. While some steps, such as weighing, adding the chemicals to the reactors, and THPC addition, are done manually in both cases, the device significantly improves several aspects of the process. In the manual method, heating is done using hot plates, which can lead to inconsistent temperature distribution between the two reactors. The device replaces this with a shared water bath, allowing both reactors to be heated under identical conditions. Stirring is also better controlled, as the device uses motor‐driven stirrers instead of magnetic stir bars. In addition, evaporation is minimized by keeping the reactors closed throughout the production process, while the manual method uses only aluminum foil to cover the containers, which is less effective and error‐prone. Furthermore, temperature is continuously monitored with a three‐channel thermometer, compared to manual insertion and removal of the thermometers with the manual method. Cooling is also made more reproducible, as ice is added to the common water bath rather than around each reactor individually, allowing for uniform and simultaneous cooling. Finally, the mixing of monomer into the gelatin solution is performed using nitrogen pressure and tubing, which reduces oxygen exposure and human handling compared to manual pouring. The same method is used to fill the gel into the final containers, which minimizes oxygen contamination and formation of air bubbles compared to the use of syringes.

**TABLE 2:** Comparison of manual PAGAT gel production method as described by Elter et al. 2021 and the new device‐assisted method together with its advantages.

Procedure	Manual preparation	Device‐assisted	Advantage of device‐assisted
**Weighing**	Manually	Manually	—
**Reactor filling**	Manually	Manually	—
**Heating**	Hot plate	Water bath	Uniform and simultaneous temperature control for both reactors
**Stirring**	Magnetic stirrer	Motor‐controlled stirrer	More consistent mixing, less manual variation
**Evaporation**	Covered with aluminum foil	Closed system with sealed lids	Reduced evaporation, improved reproducibility
**Temperature**	Single thermometer, manual readout	Three‐channel electronic monitoring	Continuous and simultaneous temperature monitoring
**Cooling**	Ice added around each reactor	Ice added to water bath	More uniform and simultaneous cooling of both reactors
**Mixing**	Manual pouring	Nitrogen pressure‐driven transfer via tubing	Less evaporation and oxygen exposure, reduced human errors
**THPC**	Manually	Manually	—
**Filling**	Manual with syringes	Nitrogen pressure‐driven filling through tubing	Reduced oxygen contamination, fewer air bubbles, improved reproducibility

## 4 DISCUSSION

This study investigated the reproducibility, stability, and dosimetric performance of PAGAT gel prepared with a new gel preparation device. The results demonstrate that the gel prepared with the developed device enables reliable gel dosimetry while highlighting critical factors influencing the dose‐response of the gel.

### 4.1 Reproducibility of gel preparation

Reproducibility is a fundamental requirement for any dosimetric system. The signal of three independently prepared batches irradiated with increasing dose levels exhibited a mean standard deviation of 1.1%, (maximum 1.8 %). The strong linear correlation (*R* 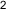 = 0.995) further validates the reliability of the proposed approach. These findings are consistent with studies by Venning et al.^8^ and Azadbakht et al.,^9^ who also reported similar R2 values and high reproducibility in PAGAT gel dosimetry when strict preparation protocols were followed.

### 4.2 Stability of PAGAT gel over time

The long‐term stability of the gel was evaluated by irradiating vials at different time points up to 14 days after preparation. No systematic signal loss was observed, and the mean standard deviation across dose levels was 3.7%. These findings suggest that the gel remains dosimetrically stable for at least two weeks after preparation. This stability is essential for practical applications where immediate irradiation may not be feasible. De Deene et al.^10^, ^11^ also demonstrated post‐irradiation stability of PAGAT gels over a period of 8 days. Future studies should extend this time frame to assess the limits of the gel's stability.

### 4.3 Influence of oxygen on gel response

Oxygen is known to affect the polymerization of polymer gels, potentially altering their response to radiation. Our results confirmed this effect, as vials prepared without the oxygen scavenger THPC exhibited no detectable dose‐dependent R2 increase. In contrast, vials containing THPC demonstrated a clear linearly increasing dose response. These findings are consistent with those of Jirasek et al.,^12^ who demonstrated that the presence of an oxygen scavenger is essential for ensuring accurate and reliable polymer gel dosimetry. Nevertheless, the influence of oxygen involves two distinct aspects. First, dissolved oxygen within the gel solution can be effectively inactivated by THPC. Second, atmospheric oxygen from the surrounding atmosphere can compromise the gel's effectiveness if vials are not completely filled without generating air bubbles. Therefore, both controlling dissolved oxygen through THPC and avoiding air bubbles during filling are essential to maintain the gel performance. Notably, as demonstrated by Khoei et al.,^13^ it is possible to add THPC up to 24 h prior to irradiation rather than during the initial gel preparation. However, this approach was associated with an overall decrease in the dose response of the gel.

### 4.4 Impact of 3D printing on gel performance

To assess the feasibility of 3D‐printed containers for gel dosimetry, different printing conditions were evaluated. While the choice of the printer (J55 vs. Objet500) did not significantly impact the gel's response, other factors played a crucial role. Specifically, vials printed with an opaque finish (use of support material) and cleaned with NaOH exhibited no increase in slope for R2. This suggests that residual chemicals from the cleaning process may alter the gel's dosimetric properties. In contrast, vials printed in glossy mode (without using support material) maintained the expected dose response. Similar findings were reported by Elter et al.,^14^ who demonstrated that VeroClear is suitable for gel containers while the presence of support material on the inner surfaces should be avoided. However, our results indicate that it is not the support material itself but rather its combination with the cleaning method that affects gel sensitivity. We hypothesize that the porous surface of the opaque vials allows residual NaOH to be trapped, which can then lead to a base‐catalyzed hydrolysis of the acrylamide, impairing the polymerization process and rendering the gel ineffective. These findings underscore the importance of optimizing both printing and post‐processing techniques to ensure the reliability of gel dosimeters in 3D‐printed containers.

### 4.5 Limitations of the device

The device is designed as a low‐cost, user‐friendly system, aiming especially at users with limited experience, which limits the degree of automation. More advanced automation features, such as automated weighing, chemical filling, or temperature‐controlled timing of process steps, were intentionally excluded to limit costs and complexity. While a fully automated, industrial device could be envisioned for the future, this would likely reduce the chance that other users will reproduce and implement this device at their institution due to the increased price.

Another potential limitation is the evaporation and condensation that can occur on the lids of the reactors during heating. However, due to the closed system design, the condensate typically drips back into the reactor. Even if a small volume is not recovered, the loss is minimal. Additionally, while a small amount of monomer solution may remain in reactor 1, this residual volume is reproducible and significantly less variable compared to manual preparation by different users.

The current cooling method relies on adding ice to the water bath, which provides rapid and inexpensive cooling. Replacing this with a precise cooling system could improve temperature control but would also raise costs and potentially extend preparation times.

Finally, the experiments conducted in this study do not exclusively assess the reproducibility of the gel preparation device itself. Rather, they encompass multiple successive steps after preparation, including storage, irradiation, and MRI scanning. Therefore, the results reflect the combined effects of the device and of several parameters included in the gel production. This has to be considered in the interpretation of the results.

## 5 CONCLUSION

The results demonstrate that PAGAT gel can be reliably prepared and used for dosimetric applications if produced in the developed gel preparation device. This closed system simplifies handling compared to manual preparation by streamlining the mixing and filling processes, thereby reducing human error. It particularly supports less experienced users by enabling straightforward operation by starting nitrogen‐driven transfer and filling by just two valves, and providing continuous temperature monitoring of both reactors and the water bath without the need to reposition sensors during preparation. The study also highlights the potential of 3D‐printed containers for customized dosimetry solutions, provided that surface interactions and cleaning methods are carefully considered. These findings contribute to the ongoing development of accurate and reproducible polymer gel dosimeters for clinical and research applications. Given the similarities in preparation processes and the sensitivity of polymer gels to temperature fluctuations and oxygen exposure, the developed device holds promise for broader applicability, potentially enabling the standardized and reproducible fabrication of a wide range of dosimetry gels beyond PAGAT gel.

## CONFLICT OF INTEREST STATEMENT

The authors declare no conflicts of interest.
